# Nutritional Status and Intestinal Parasite in School Age Children: A Comparative Cross-Sectional Study

**DOI:** 10.1155/2016/1962128

**Published:** 2016-08-30

**Authors:** Berhanu Elfu Feleke

**Affiliations:** Department of Epidemiology and Biostatistics, University of Bahir Dar, Bahir Dar, Ethiopia

## Abstract

*Background*. The objectives of this study were to determine the burden of underweight and intestinal parasitic infection in the urban and rural elementary school children.* Methods*. A comparative cross-sectional study design was conducted. Binary logistic regression was used to identify the determinants of malnutrition or intestinal parasites. Two independent samples' *t*-test was used to identify the effect of malnutrition on school performance or hemoglobin level.* Results*. A total of 2372 students were included. Quarters (24.8%) of school children were underweight. Underweight was associated with sex [adjusted odds ratio (AOR) 0.61; 95% CI = 0.47–0.78], age [AOR = 0.21; 95% CI = 0.16–0.28], intestinal parasitic infection [AOR 2.67; 95% CI = 2–3.55], and family size [AOR 23; 95% CI = 17.67–30.02]. The prevalence of intestinal parasite among school children was 61.7% [95% CI = 60%–64%]. Shoe wearing practice [AOR 0.71; 95% CI = 0.58–0.87], personal hygiene [AOR 0.8; 95% CI = 0.65–0.99], availability of latrine [AOR 0.34; 95% CI = 0.27–0.44], age [AOR 0.58; 95% CI = 0.48–0.7], habit of eating raw vegetables [AOR 3.71; 95% CI = 3.01–4.46], and family size [AOR 1.96; 95% CI = 1.57–2.45] were the predictors of intestinal parasitic infection.

## 1. Introduction

Malnutrition is the cellular imbalance between supply of nutrients, energy, and the body demand for them to ensure growth, maintenance, and specific functions [1]. Underweight is a malnutrition stage in which weight for age of the child is less than 2 SD values (standard deviation) of the WHO (World Health Organization) child growth standards median. It indicates both acute and chronic malnutrition [2]. Globally, more than 870 million people are undernourished and 852 million of them were in developing countries [3]. More than 25% of children in developing countries were underweight, and 50% of children deaths were as a result of their poor nutritional status [4, 5].

Intestinal parasitic infections were one of the 17th neglected tropical diseases listed by the World Health Organization. Neglected tropical diseases accounts for the top 4th leading cause of communicable diseases and constituted 46–57 million disability adjusted life years lost [4, 5]. The common features of neglected tropical diseases are a proxy to poverty and disadvantage, affect populations with low visibility and political voice, cause stigma and discrimination, have an important impact on morbidity and mortality, neglected by researcher, and can be controlled effectively with low cost [6].

Worldwide, approximately more than 2 billion people are infected with helmets infection [4, 7–11]. In Africa more than 173 million people are infected with* Ascaris lumbricoides*, more than 162 million cases of* trichiasis*, and more than 198 million cases of* hook worm* [6, 12–14]. In developing countries 12% of the global disease burdens due to intestinal worm were estimated to occur on children 5–14 years old [15].

Intestinal parasitic infections were the leading cause of infection in sub-Saharan Africa resulting in avoidable death. Soil transmitted helmenths infection constitutes 80% of the neglected tropical disease burden [4, 16–22]. The effect of intestinal parasites was not limited to morbidity and mortality; their impact extends to cognitive impairment, malnutrition, anemia, decreased school attendance, increased susceptibility of the host for infections like HIV/AIDS and tuberculosis, and impairing the growth of the child and adult productivity. They also impose serious socioeconomic impact on the development of a nation [4, 9, 11, 17, 23–28].

Less emphasis was given to intestinal parasite prevention and control by decision makers. Intervention against intestinal parasitic infection can be implemented with low cost, their treatment cost less than 1 dollar per case [4, 11]. Intervention against the parasitic disease was among the cost effective intervention; for every dollar spent on deworming the society will gain more than 30 dollars [29].

Nutritional status and intestinal parasitic infections were assessed only for children less than five years of age. There are no data that can show the nutritional status and intestinal parasitic infection of children above 5 years of age, so this study was conducted to fill these gaps. The effects of nutritional status or intestinal parasite were mainly studied on the morbidity and mortality of the children and this study will increase the intersectoral collaboration of decision makers in the area of health and education by showing the effect of nutritional status or intestinal parasite on the school performance of children. Usually both intestinal parasitic infections and malnutrition were studied in rural setting and their burden on the urban areas was not properly assessed, so this study will answer the question that malnutrition or intestinal parasites were the problem of urban area or not.

## 2. Objectives 

The objectives of this study were

- to describe the burden of malnutrition among school children in urban and rural setting;
- to describe the burden of intestinal parasitic infection among school children in urban and rural setting;
- to identify the determinants of malnutrition and intestinal parasitic infection in urban and rural setting;
- to identify the effect of malnutrition and intestinal parasitic infection on the school performance of children.

## 3. Methods 

A comparative cross-sectional study design was implemented in urban and rural settings. The study was conducted in the city of Bahir Dar (urban setting) and Mecha District (rural setting). Bahir Dar City was located 578 kilometers northwest of Addis Ababa (the capital city of Ethiopia). Bahir Dar City contains 37 elementary schools with a total of 45,740 students. Also the city contains 10 governmental health centers, one government hospital, one private hospital, and more than 20 private clinics. Mecha District is a rural area located 40 km to the north of Bahir Dar with 40 kebeles (the smallest administrative unit in Ethiopia). The district contains 101 elementary schools and 80,727 children in these elementary schools. The district contains 10 governmental health centers and 26 health posts.

The target population was all elementary school age children. The study population was selected elementary school students from Bahir Dar City and Mecha District. Students that received anthelmentic medication in the past 4 weeks or students unable to give stool and blood samples were excluded. The sample size was calculated using Epi-info software version 7 assuming 95% CI, 90% power, 50% malnutrition or intestinal parasitic infection in rural children, odds ratio of 1.5, design effect of 1.8, a rural to an urban ratio of 2, and a 15% nonresponse rate. The final sample size was 2,509 students. A multistage sampling technique was used to select the school children. For Bahir Dar, first 10 elementary schools were selected from a total of 37 elementary schools using the lottery method. Then, simple random sampling technique was used to select children from the 10 schools. For Mecha District, first 9 kebeles were selected from a total of 40 cables using the lottery method, one elementary school was selected from each kebele by simple random sampling using lottery method, and finally the required number of children were selected from the 9 schools by simple random sampling technique. The data were collected from March to May, 2014.

The data collection process contains 3 parts: interviewing the parents/guardians of the children, measuring anthropometric indicators, and collecting the stool and blood samples from the children.

For the interview part first the questioner was prepared in English then translated to Amharic (local language) then back to English to keep its consistency. The interview was conducted by 25 diploma nurse professionals and supervised by 5 degree holder health professionals. School performance was measured by using their previous semester CGPA (cumulative grade point average) of students.

### 3.1. Anthropometry

Weight and height of each child were measured by a clinical nurse. Digital weight scale was used to measure the weight of each child and weight was measured to the nearest 0.1 kilograms. Height was measured using the vertical measuring rod to the nearest 0.1 cms. The child's shoes, hair clips, and braids were removed before measurement, children were positioned feet together flat on the ground, heels touching the back plate of the measuring instrument, legs straight, buttocks against the backboard, scapula against the backboard, and arms were loosely at their side.

### 3.2. Blood and Stool Samples

The blood and stool samples were collected by 8 first degree holder laboratory technologists and supervised by 2 second degree holder laboratory technologists. From each student one gram stool sample was collected in 10 mL SAF (Sodium Acetate-Acetic Acid-Formalin) solution. A concentration technique was used. The stool sample was well mixed and filtered using a funnel with gauze and then centrifuged for one minute at 2000 RPM (Revolution Per Minute) and the supernatant was discarded. 7 ML (Milliliter) normal saline was added and mixed with a wooden stick, 3 ML ether was added and mixed well then centrifuged for 5 minutes at 2000 RPM. Finally the supernatant was discarded and the whole sediment was examined for parasites [30]. 1 ML blood sample was collected from each child following standard operational procedures to measure the hemoglobin level of children using Mindray hematology analyzer.

To maintain the quality of the data pretest was conducted with 50 parents, training was given for data collectors and supervisors and the whole data collection process were closely supervised by the investigator and supervisors. The collected data were checked for its completeness. The data were entered in the computer using Epi-info software by 10 IT (Information Technology) technicians and analyzed using SPSS software version 20 and WHO Anthro-plus software.

To identify the prevalence of intestinal parasites or nutritional status of the children descriptive statistics were used. Binary logistic regression was used to identify the determinants of malnutrition or intestinal parasites. Variables with *p* value less than 0.05 and 95% CI were used to discover the determinants of nutritional status or intestinal parasite. Two independent samples' *t*-test was used to identify the effect of malnutrition on school performance or hemoglobin level.

## 4. Ethics Statement

Ethical clearance was granted from Amhara National Regional State Health Bureau ethical committee. Permission was obtained from the school directors; written informed consent was obtained from each study's participants; for minors, written informed consent was obtained from parents or guardians of the children. The confidentiality of the data was kept at all steps. Study participants right to withdraw from the study at any point was respected. Students with intestinal parasites or low hemoglobin counts were referred to the nearby health center for further management.

## 5. Results 

### 5.1. Population Profile

A total of 2372 students was included giving a response rate of 94.54%. The mean age of the students was 12.75 years (standard deviation (SD) = 2.92 years). Males constituted 51.4% (1220) of the study participants. The mean height of the students was 151.2 centimeters (SD = 16.17 CM). The mean weight of the students was 41.32 KG (SD = 14.1 KG) (Table 1).

## 6. Malnutrition (Underweight)

A quarter of school children (24.8%, 95% CI [23%–26%]) were malnourished and 10.8% of school children were severely malnourished. After adjusting for age, sex, intestinal parasite infection, residence, mother education, average monthly income, availability of latrine, parent work, and family size, malnutrition was associated with sex of the child, age, intestinal parasitic infection, and family size (Table 2).

The prevalence of underweight in female school age children was 21.96% and the prevalence of underweight in male age children was 27.13%. This result was statistically significant (*p* value less than 0.01). Being of female gender decreases the risk of underweight by 39% [AOR 0.61; 95% CI = 0.47–0.78]. Availability of latrine decreases the risk of underweight by 36% [AOR 0.64; 95% CI = 0.48–0.85]. The prevalence of underweight in school children whose age was greater than or equal to 13 years was 11.67%, whereas prevalence of underweight in children whose age was less than 13 years was 33.24%. This result was statistically significant (*p* value < 0.01). School children whose age is greater than or equal to 13 years were 79% protected from being underweight as compared to children whose age was less than 13 years [AOR 0.21; 95% CI = 0.16–0.28]. The odds of underweight in school children with intestinal parasitic infection increased 2.67-fold as compared to children with no intestinal parasitic infection [95% CI = 2.00–3.55]. Family size directly affects the nutritional status of children; the odds of underweight in school children with family size greater than 4 were 23-fold higher as compared to children with family size less than or equal to 4 [95% CI = 17.67–30.02]. The burden of malnutrition on urban and rural school children was the same.

### 6.1. Intestinal Parasitosis

The prevalence of intestinal parasites was 61.7% (95% CI = 60%–64%).* Hookworm* was the predominant intestinal parasite followed by* Ascaris lumbricoides*. Mixed infections were found in 3.4% of school children.

After adjusting for age, sex, residence, mother's education, source of water, average monthly income, parent's work, shoes wearing practice, hand washing practices, personal hygiene, availability of latrine, swimming habits, habit of eating raw vegetables, and family size, it is found that shoe wearing practice, personal hygiene, availability of latrine, age, habit of eating raw vegetables, and family size were the predictors of intestinal parasitic infection (Table 3).

The odds of intestinal parasitic infection's for children that did not wear shoe increased by 29% [AOR 0.71; 95% CI 0.58–0.87]. The prevalence of intestinal parasitic infections in school age children that keep their personal hygiene was 53.19%, the prevalence of intestinal parasitic infections in school age children that did not keep their personal hygiene was 67.57%. This result was statistically significant [AOR 0.8; 95% CI = 0.65–0.99]. Availability of latrine decreased the risk of intestinal parasitic infections by 66% [AOR, 0.34: 95% CI = 0.27–0.44]. The odds of intestinal parasitic infections among school children that had a habit of ingesting raw vegetables increased 3.71-fold [95% CI = 3.01–4.46]. The risk of intestinal parasitic infections decreased by 42% for children whose age were greater than or equal to 13 years [AOR 0.58; 95% = 0.48–0.7]. High family size increases the risk of intestinal parasitic infection, the odds of intestinal parasitic infection for high family sized were 2-fold higher than children with low family size [AOR 1.96; 95% CI = 1.57–2.45].

### 6.2. Effect of Malnutrition

Two independent samples' *t*-test verified that malnutrition directly affects the school performance of children (Table 4).

A two independent samples' *t*-test verified that malnutrition decreased the hemoglobin count of school children (Table 5).

## 7. Discussion

Underweight is common in school children with a prevalence of 24.8% (95% CI [23%–26%]). This result agrees with findings from northern part of Ethiopia [31–39], higher than finding from west Ethiopia [40]. This is due to improper implementation of effective community health intervention.

The odds of underweight in females were 39% lower than males [AOR 0.61; 95% CI = 0.47–0.78]. This result agrees with finding from different scholars [32, 39, 41–43]. This is due to different physiological demand of nutrition for these genders. This might also be due to the reason of the different distribution of morbidity across male gender.

Availability of latrine decreases the risk of underweight by 36% [AOR 0.64; 95% CI = 0.48–0.85]. This is due to the fact that latrine utilization prevents the occurrence of intestinal parasite and decreases the occurrence of morbidity associated with intestinal parasite. Also the availability of latrine acts as a signal for the proper implementation of community health preventive activities.

The odds of underweight in school children with intestinal parasitic infection increased 2.67-fold as compared to children with no intestinal parasitic infection [95% CI = 2.00–3.55]. This finding is different from findings from different parts of the world [17, 18, 31]. This is due to the reason that intestinal parasitic infection competes for the nutritional intake of children and intestinal parasitic infection also impairs the immune system of the host so that it makes them susceptible to many diseases [25].

The odds of underweight in school children with large family size were 23-fold higher as compared to children with small family size less [95% CI = 17.67–30.02]. This finding agrees with finding from Gondar [32]. This is due to the reason that large family size will share the limited available nutrients inside that house so that children will not get the required nutritional demand for their growth.

The burden of malnutrition was the same on urban and rural elementary school children. Effective community health intervention should be implemented in urban and rural setting.

The prevalence of intestinal parasite was 61.7% (95% CI = 60%–64%). This magnitude was lower when compared to finding from Malaysia and Philippines [44, 45] and higher than finding from Cameron and eastern part of Ethiopia [40, 46]. This might be due to the different implementation strategy of primary healthcare components in these areas.

Personal hygiene greatly reduces the burden of intestinal parasites. The prevalence of intestinal parasitic infection in school age children that keep their personal hygiene was 53.19%, and the prevalence of intestinal parasitic infection in school age children that did not keep their personal hygiene was 67.57%. This result was statistically significant [AOR 0.8; 95% CI = 0.65–0.99]. This finding agrees with finding from different parts of the world [47]. This is due to the reason that proper personal hygiene breaks the chain of intestinal parasite transmission.

The odds of intestinal parasitic infection in the presence of latrine decreases by 66% [AOR 0.34; 95% CI = 0.27–0.44]. This finding agrees with finding from different parts of the world [32]. This is due to the fact that latrine will dump all the intestinal parasites away from the susceptible host.

The odds of intestinal parasitic infection among school children that had a habit of ingesting raw vegetables increased 3.71-fold [95% CI = 3.01–4.46]. This finding agrees with finding from Delgi [48]. This is due to the fact that raw vegetables are good culture media for intestinal parasites.

The burden of intestinal parasitic infection was higher in early age. The risk of intestinal parasitic infection decreased by 42% for children whose age is greater than or equal to 13 years [AOR 0.58; 95% = 0.48–0.7]. This finding agrees with finding from different parts of the world [4]. This is due to the reason that as the age of children increases, they can protect themselves from intestinal parasitic infection because they are active.

The odds of intestinal parasitic infection for high family size children were 2-fold higher than children with low family size [AOR 1.96; 95% CI = 1.57–2.45]. This is due to the reason that overcrowding decreases the probability of latrine utilization and personal and environmental hygiene will not be kept if the size of family is large.

The burden of intestinal parasitic infection on urban and rural elementary school students was the same, calling for proper implementation of primary healthcare components in urban and rural areas.

Malnutrition decreases the school performance of children by 1.32%. Improving the nutritional status of school children increases the quality of education. This finding agrees with finding from different parts of the world [4, 8, 9]. This is due to the reason that proper nutritional intake increases the brain cell development and improves the cognitive performance of the child. Malnutrition also decreases the hemoglobin level of school children by 0.1 mg/dL making the school children anemic. This is due to the reason that malnourished children will not take enough quantity of iron per the demand of the child.

## 8. Conclusions

Malnutrition (underweight) was higher in urban and rural elementary school students. The nutritional status of children was affected by gender, intestinal parasitic infection, age of the child, family size, and latrine utilization. High prevalence of intestinal parasitic infection was observed in urban and rural elementary school students.

## Figures and Tables

**Table 1:** Population profile of the study participants (*n* = 2372).

SN	Population profile	Frequency	Percentage
(1)	*Age (in years)*		
	5–9	384	16.2
	10–14	1296	54.6
	15–19	692	29.2

(2)	*Sex*		
	Male	1220	51.4
	Female	1152	48.6

(3)	*Height (in CM)*		
	103–122	130	5.5
	123–142	511	21.5
	143–162	1021	43
	163–182	660	27.8
	>182	50	2.1

(4)	*Weight (in KG)*		
	15–34	963	40.6
	35–54	942	39.7
	55–74	460	19.4
	75–94	7	0.3

(5)	*Grade*		
	0–4	423	17.8
	5–8	1949	82.2

(6)	*Residence*		
	Rural	1578	66.5
	Urban	794	33.5

(7)	*Source of water*		
	Pipe	1459	61.5
	Other	913	38.5

(8)	*Income per month*		
	<1000 birrs	1598	67.4
	≥1000 birrs	774	32.6

(9)	*Availability of latrine*		
	Available	1805	76.1
	Not available	567	23.9

(10)	*Work of parent*		
	Farmer	1344	56.7
	Other	1028	43.3

**Table 2:** Predictors of underweight in school age children (*n* = 2372).

Variable	Underweight		COR (95% CI)	AOR (95% CI)	*p* value
	Yes	No			
*Sex*			0.79 [0.62–0.92]	0.61 [0.47–0.78]	<0.01
Female	253	899			
Male	331	889			

*Latrine*			0.61 [0.5–0.76]	0.64 [0.48–0.85]	<0.01
Available	403	1402			
Not available	181	386			

*Age*			0.26 [0.2–0.32]	0.21 [0.16–0.28]	<0.01
≥13	116	878			
<13	468	910			

*Intestinal parasitic infection *			3.34 [2.65–4.22]	2.67 [2–3.55]	<0.01
Yes	471	992			
No	113	796			

*Family size*			19 [14.98–24.11]	23 [17.67–30.02]	<0.01
>4	407	193			
≤4	177	1595			

**Table 3:** The determinants of intestinal parasite in school age children.

Variables	Intestinal parasite		COR [95% CI]	AOR [95% CI]	*p* value
	Yes	No			
*Shoe wearing practice*			0.52 [0.44–0.62]	0.71 [0.58–0.87]	<0.01
Yes	560	493			
No	903	416			

*Personal hygiene*			0.55 [0.46–0.65]	0.8 [0.65–0.99]	0.04
Kept	517	455			
Not kept	946	454			

*Availability of latrine*			0.3 [0.24–0.38]	0.34 [0.27–0.44]	<0.01
Yes	1005	800			
No	458	109			

*Ingesting raw vegetables*			3.8 [3.18–4.54]	3.71 [3.01–4.46]	<0.01
Yes	1044	360			
No	419	549			

*Age*			0.53 [0.44–0.62]	0.58 [0.48–0.7]	<0.01
≥13	525	469			
<13	938	440			

*Family size*			2.06 [1.67–2.54]	1.96 [1.57–2.45]	<0.01
>4	442	158			
≤4	1021	751			

**Table 4:** School performance and malnutrition.

Group	Average of previous semester	Mean difference [95% CI]	*t*	Degree of freedom	*p* value
Not undernourished	69.53	1.32 [0.05–2.28]	2	2370	0.04^*∗*^
Undernourished	68.22				

^*∗*^Equal variances assumed.

**Table 5:** Hemoglobin count and malnutrition.

Group	Average hemoglobin count	Mean difference [95% CI]	*t*	Degree of freedom	*p* value
Not undernourished	11.65	0.1 [0.03–0.16]	2.78	2370	<0.01^*∗*^
Undernourished	11.56				

^*∗*^Equal variances assumed.

## Additional Points

*Recommendation*. High level decision makers in the area of health should give special emphasis to the nutritional intervention of the child and on averting the risk of intestinal parasitic infection. Decision makers in the area of child education should include nutritional intervention as a mechanism to increase the quality of child education. Institutions that work in the area of malnutrition should include family planning as one form of keeping the child well nourished.
